# What Are the Minimal Feeds Required for Starting Enteral Ibuprofen in Preterm Infants with PDA?

**DOI:** 10.3390/pediatric14030040

**Published:** 2022-07-24

**Authors:** Aimann Surak, Abbas Hyderi

**Affiliations:** Northern Alberta Neonatal Program, Philip C. Etches NICU, Royal Alexandra Hospital Site, DTC 5027, 10240 Kingsway NW, Edmonton, AB T5H 3V9, Canada; abbas.hyderi@albertahealthservices.ca

**Keywords:** PDA, preterm, ibuprofen

## Abstract

Ibuprofen is commonly used for the treatment of hemodynamically significant patent ductus arteriosus (PDA) in preterm infants. It seems that the oral formulation incurs a higher closure rate and has a better safety profile in preterm infants born > 26 weeks’ gestation. There is no consensus across Canadian centers regarding the minimum volume of enteral feeds required prior to starting ibuprofen for the treatment of patent ductus arteriosus, and the current practice is comfort-based depending on the centre and the local prevalence of neonatal morbidities.

## 1. Key Points

This brief review highlights the lack of knowledge and variation in clinical practice in terms of the minimum feeds required prior to starting enteral ibuprofen for PDA treatment.

## 2. Introduction

It is well known that that non-steroidal anti-inflammatory agents (NSAIDs) inhibit arachidonic acid-forming prostaglandin substrates. Ibuprofen inhibits both cyclooygease-1 and cyclooxygenase-2, which are enzymes necessary for the conversion of arachidonic acid to various prostaglandins. Among them, prostaglandin E2 is the most potent vasodilator of the ductus. This action will cause the constriction of the patent ductus arteriosus (PDA), and ibuprofen is commonly used for that purpose [1]. The current Standard Ibuprofen dosing is 10 mg/kg/dose and 5 mg/kg/dose every 24 h (a 3-day course), administered enterally or parenterally [1].

We performed a quick audit survey between the Canadian centres asking a simple question regarding the minimal volume of enteral feeds required so that the clinical team feels comfortable initiating the oral ibuprofen therapy for hemodynamically significant PDA. As expected, there was significant variation from as low as trophic feeds (10–20 mL/kg/day) to as high as 120 mL/kg day.

## 3. Literature about Ibuprofen Dose

Oral Ibuprofen is superior to IV Ibuprofen for closing the PDA in preterm infants, with an RR of 0.38 (CI 0.26–0.56) and moderate grade of evidence [1]. This systematic review analyzed five non-blinded RCTs [2,3,4,5,6]. The different demographics prevent generalizability and applicability. Oral Ibuprofen was given between 48 and 96 h of life after the exclusion criteria were met. Only two of the five RCTs provided information on ibuprofen osmolality [5,6].

Erdeve et al. performed an RCT comparing oral versus IV Ibuprofen in 80 preterm infants born at 26 weeks’ gestation and with a birth weight of 862 g at 48–96 h of age while being enterally fed 20–40 mL/kg/day. The ibuprofen used was standard dosing, and the osmolality was 312 mosm/L (Pedifen, Atafarm, Turkey). The researchers concluded that oral ibuprofen had an 84% success rate in closure compared to IV with a 62% success rate; additionally, the oral form had no pulmonary hemorrhage, whereas the IV Ibuprofen did present pulmonary hemorrhaging in 9% of cases. Moreover, gastrointestinal side effects, including necrotizing enterocolitis (NEC) and spontaneous intestinal perforation (SIP), were lower in the oral ibuprofen group, although they did not meet statistical significance [5].

Gokmen et al. conducted an RCT comparing oral versus IV ibuprofen in 100 VLBW preterm infants at about 2–4 days of age. There was a higher ductal rate in the oral ibuprofen group that used standard dosing; the osmolality was 312 mosm/L (Pedifen, Atafarm, Turkey) [6].

Oncel et al. conducted an RCT comparing oral ibuprofen versus oral acetaminophen in 90 preterm infants born between 26–27 weeks’ gestation with a median birth weight of 973 g. The oral ibuprofen group was given the standard dosing (Pedifen, Atafarm, Turkey), with an osmolality of 312 mosm/kg. The participants received therapy between 48–96 h of life at an enteral feed volume of 20–40 mL/kg/day. There was a higher ductal closure rate in this group with no difference in the NEC (5% vs. 7%) or GI side effects profile between groups [7].

In another recent RCT, Kumar et al. compared oral ibuprofen to oral acetaminophen in 160 preterm infants born at approximately 28 weeks’ gestation (22% of the cohort were less than 28 weeks’ gestation). Feeding protocol and osmolality of ibuprofen (Ibugesic, Cipla, India) information was not available. The researchers did not find either agent to be superior for closing PDA [8].

Ghanem et al. conducted an RCT with 60 preterm infants born at 29 weeks’ gestation, comparing a placebo to a standard dose of oral ibuprofen (osmolality 320 mosm/L with PH 5.5; name not given). The authors found no increased GI side effects in the ibuprofen group with an excellent ductal closure rate of ~90% [9].

A higher dose of ibuprofen was compared to the standard dose in an RCT by Pourarian et al. in preterm infants born at 31 weeks’ gestation with birth weights of 1.4 kg on 3rd–7th days of life. There was no difference in the GI side effects profile or NEC between the two groups [10].

## 4. Pharmacokinetics Data

Barzilay et al. studied the pharmacokinetics of 13 extreme preterm infants born at 27 weeks’ gestation with birth weights of ~1000 g and used oral ibuprofen (Nurofen, Reckitt Benckiser, Israel) at a standard dosing with osmolality of 320 mosm/L. The area under curve (AUC) was higher with oral Ibuprofen compared to IV Ibuprofen-Lysine’s pharmacokinetics from Hirt et al.’s dataset. The AUC (0–24) was high at about 618 mg/mL*h [11].

Hirt et al. conducted their study in babies born at ~28 weeks’ gestation and suggested that an AUC (0–24) > 600 mg/mL*h would have a PDA closure rate of 91%. The oral Ibuprofen likely had a slower absorption time and longer contact time and hence achieved a greater PDA closure rate [12].

Sharma et al. studied the pharmacokinetics of oral Ibuprofen (20 mg/mL Ibugesic, Cipla, India) in 20 VLBW infants and concluded that the time to maximum concentration was 2–3 h and the half-life was ~16 h with an AUC of about 400 +/− 80. The renal elimination was biphasic. Interestingly, the authors concluded no correlation with the elimination half-lives and gestation ages [13].

Hence, currently, the best evidence available is for gestations of greater than 26 weeks [5]. In our literature search, we were not able to find matching cohorts for preterm infants born < 25 weeks’ gestation.

## 5. Osmolality and GI Morbidity/NEC

Historical nutrition standards have been established since 1976 by the AAP Committee on Nutrition.

The standards suggested a maximum of 450 mosm/kg osmolality in feeds across all newborns. A higher osmolality was associated with a higher incidence of delayed gastric emptying, leading to longer stasis and NEC. However, no evidence was provided on the rationale of choosing such a maximum limit [14].

Nevertheless, this has remained the nutrition standard of maximum osmolality for nearly half a century.

In 2019, Ellis et al. performed a systematic review of 618 infants across 10 studies. The population ranged from 600–2000 g and gestational ages of 24–33 weeks. The authors found no consistent evidence that a feed osmolality of 300–500 mosm/kg poses any safety risk or causes any adverse gastrointestinal events, especially feeding intolerance, except at very high levels (e.g., >539 mosm/L) [15].

Interestingly, the osmolality of medications and nutrition may be treated as two separate molecules in the neonatal gut. Drugs often have carrier molecules that can diffuse across membranes, whereas nutrients in feeds do not readily diffuse. Therefore, hyperosmolar feeds are likely to pose a higher osmolar risk than hyperosmolar dugs. Professor Tanis Fenton (at the University of Calgary), a renowned neonatal nutrition expert, stated in a commentary “the only substance that needs to be considered risky in terms of osmolality for potential inducement of NEC are those that create an osmotic gradient across the intestinal membrane in vivo. Some of the measured drugs may not pose this risk of osmolar load” [16].

The osmolality of a specific compound is quite variable, as it depends on many factors, such as the concentration and manufacturer. For instance, even the same medication with the same brand and same manufacturer has different osmolality data. For example, Motrin (40 mg/mL, McNeil) is 2037 mosm/kg, according to Da Silva et al., whereas for Leong et al. it was 1775 mosm/kg. This variation is perhaps also related to different osmometer/techniques or its excipient/preservatives, among other explanations. Furthermore, other investigators have often suggested the simplest way to deal with high osmolar medications is to dilute the medications to iso osmolar status. However, since neonatal medications remain at a higher osmolality (greater than the AAP limit) even after dilution, Radmacher et al. suggested caution and an evaluation of the co-administration of milk products as a potential contributor to the feed intolerance of the preterm infant [17].

In general, the management of enteral feeds in preterm infants with a hemodynamically significant PDA remains a major challenge. The efficacy of oral ibuprofen is higher the earlier it is used and potentially corresponds to more side effects; however, there is a gap in the literature, with no clearcut answers regarding the timing of when the oral ibuprofen incurs the highest closure rate and whether it has a better safety profile in preterm infants [18].

## 6. The Ibuprofen Network Metanalysis

Mitra et al. recently published an interesting network meta-analysis of over 4200 infants across 60 RCTs with PDA closure outcomes among various pharmacotherapies [19], where a heat map ranking system was used to analyze pharmacotherapy using SUCRA methodology. In the analysis of this ranking system, the standard-dose oral Ibuprofen (0.71) had the best outcome for mortality and BPD (0.87); however, a high dose of oral Ibuprofen was superior for PDA closure and the prevention ligation (0.98), although it had higher oliguria and mortality.

## 7. Conclusions

The advantages of oral ibuprofen compared to IV formulation can be summarized by its higher efficacy for ductal closure, lower cost, and more favourable safety profile. This is more obvious in preterm infants born at >26 weeks’ gestation. However, for the preterm infants born at <26 week’ gestation, safety, pharmacokinetics, or dynamics data are not directly available. The nearest extrapolation would be Erdeve et al.’s and Gokmen et al.’s RCTs [5,6]. Both trials used oral standard dose ibuprofen (312 mosm/kg) where the feeds were only 20–40 mL/kg/day by 48–96 h of life and reported higher ductal closure rates with no increased GI morbidity. There is no consensus across Canadian centres regarding the minimum volume of enteral feeds required prior to starting ibuprofen for the treatment of patent ductus arteriosus, and the current practice is comfort-based depending on the centre and the local prevalence of neonatal morbidities. In general, there are limited data and the literature is lacking on preterm infants born < 26 weeks; therefore, future trials and studies are needed.

## Abbreviations

AUC	Area under the curve
ELBW	Extreme low birth weight
IV	Intravenous
NEC	Necrotizing enterocolitis
NSAIDs	Non-steroidal anti-inflammatory agents
PDA	Patent ductus arteriosus
RCT	Randomized controlled trial
SIP	Spontaneous intestinal perforation
VLBW	Very low birth weight
